# Metabolic Pathways and Potencies of New Fentanyl Analogs

**DOI:** 10.3389/fphar.2019.00238

**Published:** 2019-04-05

**Authors:** Maurice Wilde, Simona Pichini, Roberta Pacifici, Adriano Tagliabracci, Francesco Paolo Busardò, Volker Auwärter, Renata Solimini

**Affiliations:** ^1^Department of Forensic Toxicology, Institute of Forensic Medicine, Medical Center – University of Freiburg, Faculty of Medicine, University of Freiburg, Freiburg im Breisgau, Germany; ^2^Hermann Staudinger Graduate School, University of Freiburg, Freiburg im Breisgau, Germany; ^3^National Centre on Addiction and Doping, Istituto Superiore di Sanità, Rome, Italy; ^4^Unit of Forensic Toxicology, Section of Legal Medicine, Department of Excellence SBSP, Università Politecnica delle Marche, Ancona, Italy

**Keywords:** novel synthetic opioids, fentanyl analogs, fentanyl biotransformations, *in vivo* and *in vitro* metabolism, metabolic profile, receptor binding affinity, toxicity

## Abstract

Up to now, little is known about the metabolic pathways of new fentanyl analogs that have recently emerged on the drug markets worldwide with high potential for producing addiction and severe adverse effects including coma and death. For some of the compounds, limited information on the metabolism has been published, however, for others so far no information is available. Considering the well characterized metabolism of the pharmaceutically used opioid fentanyl and the so far available data, the metabolism of the new fentanyl analogs can be anticipated to generally involve reactions like hydrolysis, hydroxylation (and further oxidation steps), *N*- and *O*-dealkylation and *O*-methylation. Furthermore, phase II metabolic reactions can be expected comprising glucuronide or sulfate conjugate formation. When analyzing blood and urine samples of acute intoxication cases or fatalities, the presence of metabolites can be crucial for confirmation of the uptake of such compounds and further interpretation. Here we present a review on the metabolic profiles of new fentanyl analogs responsible for a growing number of severe and fatal intoxications in the United States, Europe, Canada, Australia, and Japan in the last years, as assessed by a systematic search of the scientific literature and official reports.

## Introduction

Opiates have been used for thousands of years to treat a broad variety of conditions. The first semi-synthetic opioids (such as heroin) were derived from the opium alkaloid morphine. In association with the discovery and deeper investigation of the opioid receptors numerous synthetic, structurally diverse opioids were developed by research chemists and pharmaceutical companies. Fentanyl has first been synthesized by Paul Janssen in 1959 (Janssen, 1965) and was derived from the synthetic opioid meperidine. Its pharmacological action is 50–100 times more potent than morphine and 25–40 times more than heroin, and it is commonly used in anesthesia and pain treatment (US Drug Enforcement Administration [US DEA], 2015; National Institute on Drug Abuse [NIDA], 2016). Fentanyl and its clinically used analogs are regarded as highly potent μ-opioid receptor agonists.

Besides the medicinal usage and progress in the therapeutic application of opioids, misuse of opioids has always been an issue. However, non-medical use of opioids often leads to health problems due to the high addictive potential of opioids and their severe acute side effects like respiratory depression. Repeated opiate and opioid use leads to tolerance, a contributing factor to opioid dependence. Development of tolerance is a controversially discussed topic and not fully understood yet. However, there is a consensus that different mechanism are involved, among them pharmacodynamic tolerance (adaptive changes in networks or pathways in organs and tissues affected by drug interaction), behavioral tolerance, pharmacokinetic (metabolic) tolerance and tachyphylaxis (Bespalov et al., 2016). In both clinical use and misuse tolerance may lead to dose escalation and finally severe adverse effects.

Over the last few years a wave of highly potent synthetic opioids emerged on the market of new psychoactive substances (NPS). These ‘new synthetic opioids’ (NSO) are often derived from fentanyl (also known as ‘designer fentanyls,’ ‘fentanyl derivatives,’ or ‘fentalogs’) and available at a cheaper cost compared to heroin (Marchei et al., 2018; Rothberg and Stith, 2018). Fentanyl analogs have recently been encountered as cutting agents in seized heroin samples, in ready-to-use preparations like nasal sprays or as ‘research chemicals’ marketed via internet shops. These drugs have caused an increasing number of acute intoxications and fatalities in North America, as well as in Europe, Japan, Canada, and Australia (Pichini et al., 2017, 2018). Plenty of pharmacokinetic studies have been published evaluating and characterizing receptor binding and potency of fentanyl (Costa et al., 1992; France et al., 1995) and its clinically relevant analogs (Henriksen et al., 2005; Volpe et al., 2011). When comparing binding constants, it has to be kept in mind that variables like type of assay, choice of competitive ligand etc. significantly impact the experimental outcome and may lead to varying values for identical compounds. In contrast, information on pharmacological data – and in particular metabolism – of non-medically used fentanyl analogs is scant, with evident difficulties in identifying the molecules in biological fluids of the consumers in order to assess consumption (Armenian et al., 2018). In addition, ratios of parent compound and metabolite concentrations can help to examine the plausibility of specific scenarios in forensic toxicology (e.g., acute vs. slow accumulative poisoning). An early study assessing the opioid-like activity of several fentanyl metabolites in a guinea pig ileum assay found that norfentanyl, 4-ANPP (4-anilino-*N*-phenethylpiperidine) and 4-anilinopiperidine (metabolites of fentanyl) were less potent than either fentanyl or morphine by several orders of magnitude (Schneider and Brune, 1986). The only metabolite showing significant activity in this study was a phenolic derivative hydroxylated at the 4-position of the phenylethyl moiety of fentanyl, the activity of which was found to lie between morphine and pethidine.

Nevertheless, some of the fentanyl analog metabolites might retain opioid activity with clinical relevance. What has been documented for fentanyl metabolism typically translates to the new designer fentanyls, which also show an extensive metabolism, however, to varying degrees. This review article summarizes the current knowledge on pharmacological data with a focus on the metabolism of novel fentanyl analogs.

## Methods

### Procedures for Assessment of Metabolic Profiles

To investigate the metabolism of a distinct compound, *in vivo* or *in vitro* approaches can be used: *in vivo* studies are performed in animals or humans, whereas *in vitro* approaches include the use of human liver microsome preparations, human hepatocytes or fungi as models for metabolism. In general, *in vivo* studies in humans would be the best choice due to limited transferability of animal data, but require ethical approval and are often not feasible. Human self-administration studies or the investigation of body fluids of death cases can serve as an alternative if available. However, such studies may show biased metabolic profiles due to health conditions or enzymatic phenotypes of study subjects. *In vitro* approaches generally do not reflect the full human metabolism, but are much easier to implement. Human hepatocytes are a commonly used model simulating human hepatic metabolism. However, due to varying factors like cell line and culture environment, the metabolic profile resulting from hepatocyte incubation may vary and does often not reflect the metabolic profile obtained *in vivo* sufficiently. Human liver microsomes or fungi like *Cunninghamella elegans* are further tools to produce *in vitro* metabolites. They are relatively easy to handle and cost-efficient, but may lack the ability to produce the whole human metabolic spectrum.

Analytical identification of metabolites is usually performed by mass spectrometric techniques like liquid chromatography-high resolution mass spectrometry (LC-HRMS) and use of different scan modes of tandem mass spectrometry. Differentiation of isomers often affords isolation of specific metabolites and nuclear magnetic resonance (NMR) spectroscopy analysis.

### Literature Search

MEDLINE for biomedical literature and EMBASE for pharmacological literature as well as multidisciplinary databases such as Scopus and Web of Science were searched using the following combined terms: fentanyl analogs or analogs or derivatives or designer fentanyls or fentalogs, fentanyl, remifentanil, sufentanil, alfentanil, acetylfentanyl, acryloylfentanyl (or acrylfentanyl), α-methylfentanyl, butyr(-yl)fentanyl, carfentanil, cyclopropylfentanyl, cyclobutyl-fentanyl, cyclopentylfentany, cyclohexylfentanyl, 2,2,3,3-tetramethylcyclopropylfentanyl, crotonylfentanyl, 4-fluoro-isobutyr(-yl)fentanyl, isofentanyl, furanylfentanyl, methoxya-cetylfentanyl, ocfentanil, ortho-fluorofentanyl, tetrahydro-furanylfentanyl, metabolism, metabolic networks, metabolic pathways, μ-opioid receptor, opioid receptor binding. Further studies were retrieved by hand search through the reference lists of the selected articles. Moreover, a search for reports was conducted on Institutional websites, to identify documentation published by international agencies or institutions including the United States Drug Enforcement Administration (US DEA), United States National Institute on Drug Abuse (NIDA), World Health Organization (WHO) and the European Monitoring Centre for Drugs and Drug Addiction (EMCDDA).

## General Remarks

### Opioid Receptors

Opioid receptors are membrane bound G-protein coupled receptors predominantly located at the synaptic complex in the central nervous system but are also found in peripheral tissues. In the 1960s and 1970s first binding studies were performed by Van Praag and Simon (1966), Ingoglia and Dole (1970), Simon et al. (1973), Terenius (1973) and Pert and Snyder (1973) locating the opioid receptors in different brain areas using radio-labeled ligand assays. First proof for the existence of multiple opioid receptors was published by Martin (1967) proposing three different types of the opioid receptor (μ, κ, and δ). The μ-opioid receptor (MOR) named by its agonist morphine is mainly located in brain tissue and the gastrointestinal (GI) tract. This receptor mediates many of the typical opiate effects like analgesia, euphoria, miosis, physical dependence, reduced GI-mobility and respiratory depression. Three subtypes of the μ-opioid receptor (μ_1_, μ_2_, and μ_3_) have been identified, while μ_1_ is characterized best (Pan et al., 2005). The κ- and δ-opioid receptors are both found primarily in the brain tissue. For the κ-receptor three subtypes and for the δ-receptors two subtypes have been identified (Rothman et al., 1989; Portoghese and Lunzer, 2003). In principal, the same central nervous effects are produced by activation of the κ receptors as for the μ receptors, but additionally κ receptor agonists can cause hallucination and dissociation. δ-Opioid receptors are believed to contribute to analgesia as well, but also modulate immune response of myenteric neurons (Poonyachoti et al., 2001). Fentanyl and its analogs have been specifically designed for the activation of the μ-opioid receptors and usually show high selectivity for this receptor type. This is one of the factors complicating a direct comparison of morphine and fentanyl potency. Considering potency regarding central nervous effects, transmission through the blood-brain barrier has to be taken into consideration, too.

## Fentanyl and Fentanyl Analogs

**Figure 1:**
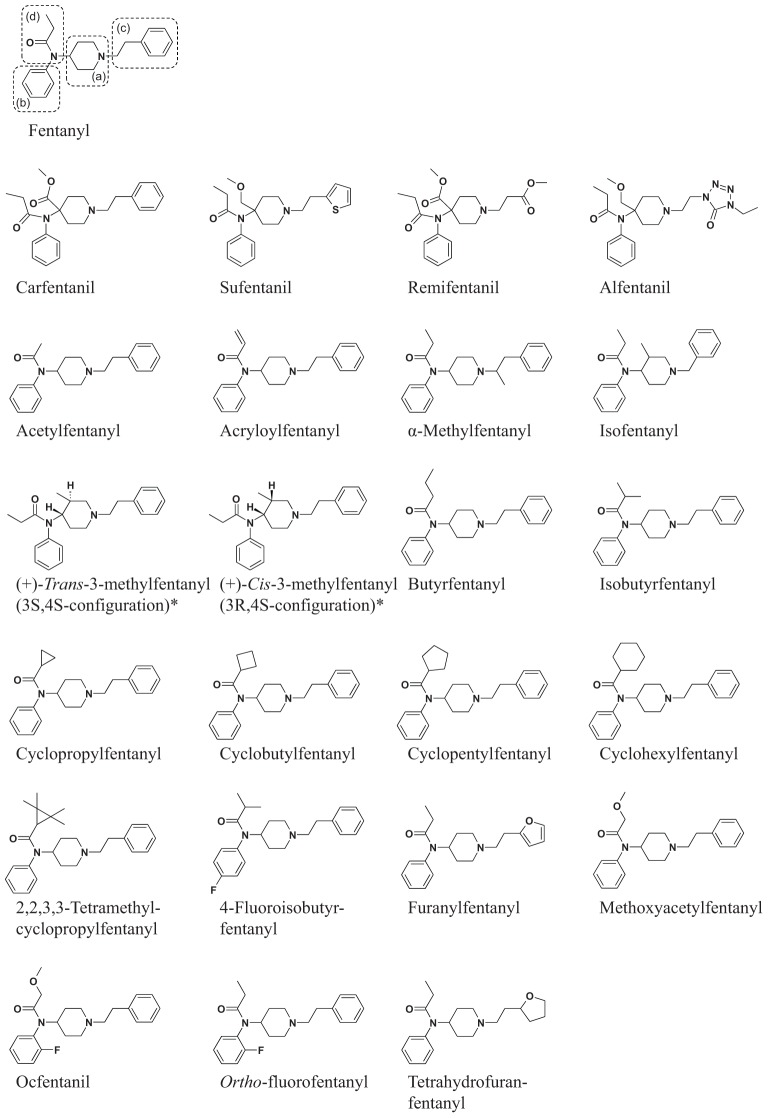
Chemical structures of fentanyl and reviewed fentanyl analogs with data on metabolism and/or potency available in the scientific literature. The structures marked with ‘^∗^’ show only one of the two enantiomers.

Fentanyl is a 2-phenylethyl-substituted 4-anilinopiperidine derivative carrying a propionylamide moiety linked to the aniline-nitrogen. In principle, there are four structural features which can potentially be modified, resulting in a huge variety of fentanyl analogs: (a) the piperidine ring, (b) the anilinophenyl ring, (c) the 2-phenylethyl substituent and (d) a carboxamide moiety linked to the anilino-nitrogen (Figure 1).

In the 1970s, Janssen Pharmaceutica patented a series of highly potent fentanyl derivatives, the *N*-4-substituted 1-(2-arylethyl)-4-piperidinyl-*N*-phenylpropanamides, such as the medically used Sufentanil and Carfentanil (Janssen, 1979). Carfentanil, which has been approved for veterinary use (Wildnil^®^) due to its extremely high potency, recently emerged as a designer drug on the recreational drug market, posing a huge health risk not only for users but also for first responders and law enforcement staff. Since the 1970s, a multitude of further analogs has been investigated (Brine et al., 1995; Wang et al., 1995; Vuckovic et al., 2009). One of the first fentanyl analogs on the designer drug market was the highly potent 3-methylfentanyl, methylated at the piperidine ring (a) (Figure 1) resulting in a pair of diastereomers (Van Bever et al., 1974). Several different substituents like halogen atoms, methyl or methoxy groups of the anilinophenyl ring (b) (Figure 1) have been published and some of these emerged on the designer drug market in recent years (United Nations Office on Drugs and Crime [UNODC], 2017). The 2-phenylethyl moiety (c) (Figure 1) substituted at the tertiary piperidinyl-nitrogen seems to improve receptor binding over non-substituted or methyl substituted compounds, presumably by fitting better into a hydrophobic cavity of the μ-opioid-receptor in close proximity to the active binding site (Jiang et al., 2000; Subramanian et al., 2000). Fentanyl analogs modified at this moiety like α-methylfentanyl have been reported to be involved in some fatal intoxication cases in the 1980s (Gillespie et al., 1982). The β-hydroxylated analog of 3-methylfentanyl, ohmefentanyl, has been well researched in the 1980s, showing extremely high potencies for some of the diastereomers (Subramanian et al., 2000). Modification of the propanamide moiety (d) (Figure 1) of fentanyl led to a huge variety of newly emerged fentanyl analogs in recent years (such as butyrfentanyl, furanylfentanyl, benzodioxole fentanyl, cyclopropylfentanyl, methoxyacetylfentanyl and many more). These derivatives are presently in the focus of research, since there is none or very little data available so far.

### Fentanyl

Fentanyl is a medically used 4-Anilinopiperidine derivative like alfentanil, sufentanil, and remifentanil. These drugs are used in surgery as adjuncts to anesthesia, for sedation and for the treatment of acute and chronic pain (Van Bever et al., 1976; Van Daele et al., 1976; Kukanich and Papich, 2009).

First metabolism studies on fentanyl were conducted by Van Wijngaarden and Soudijn (1968) monitoring the parent compound and metabolites of radio-labeled fentanyl in urine and feces of rats after intravenous administration. In the late 1980s, Banks and Ferguson (1988) described fentanyl metabolism, indicating that several factors have to be taken into consideration in order to determine drug metabolism: administration routes (intravenous, subcutaneous, transdermal, transmucosal, and spinal), tissue chosen for analysis, isolation procedure and inter- and intra-individual variation that can influence metabolite formation and distribution (Streisand et al., 1991; Solassol et al., 2005).

Fentanyl (*N*-phenyl-*N*-[1-(2-phenethyl)-4-piperidinyl]pro-panamide) has several sites for metabolic transformation. It is a heterocyclic tertiary aliphatic amine containing two different phenyl rings and an aromatic amide function. Tertiary aliphatic amines are biotransformed through a reversible reaction into tertiary amine oxides. The tertiary amines also undergo *N*-dealkylation through the carbinolamine. When this process happens on the phenylethyl side chain, in addition to the secondary amine a phenylacetaldehyde is produced, which immediately oxidizes into phenylacetic acid. Oxidation at the 2-position of the piperidine ring generates a carbinolamine, which transforms into a more stable aminoaldehyde, resulting in ring cleavage. Aromatic rings undergo oxidation producing the equivalent phenolic derivatives. Furthermore, benzylic positions are more prone to oxidation. Amide functions usually undergo hydrolysis, and oxidation of the carbon chain is also frequent (Goromaru et al., 1984; Banks and Ferguson, 1988; Vardanyan and Hruby, 2014).

**Figure 2:**
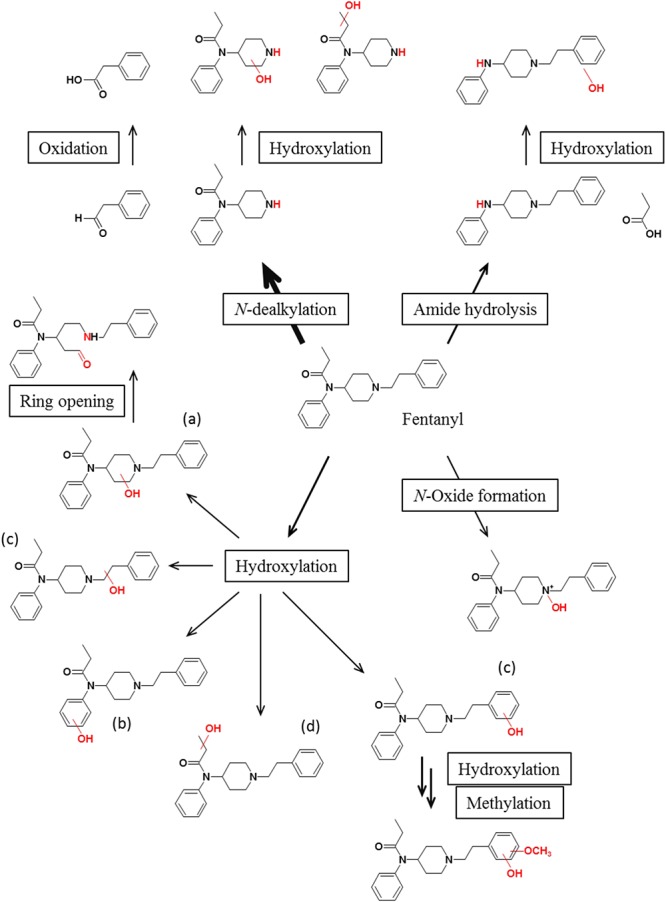
Schematic metabolic profile of fentanyl in humans, depicting the main biotransformations described in the literature. Main metabolic pathways are marked by bold arrows.

In humans, fentanyl is mainly metabolized in the liver by CYP3A4 into norfentanyl through oxidative *N*-dealkylation at the piperidine ring by hepatic CYP3A4 and 3A5 isoenzymes, which is the principal pathway of metabolism (Feierman and Lasker, 1996; Guitton et al., 1997; Labroo et al., 1997; Gudin, 2012; Bista et al., 2014; Armenian et al., 2018). The inactive metabolites and less than 10% of the intact molecule are mainly excreted in urine and feces (Mercadante, 2015; Kuip et al., 2017; Armenian et al., 2018) and less than 1% is metabolized by alkyl hydroxylation, combined *N*-dealkylation and hydroxylation or amide hydrolysis to the inactive compounds hydroxyfentanyl, hydroxy norfentanyl, and despropionylfentanyl (Kuip et al., 2017; Wu et al., 2017). The schematic human metabolic profile of fentanyl is depicted in Figure 2.

Fentanyl is also metabolized to norfentanyl in human duodenal microsomes; the mean rate is approximately half of the hepatic metabolism. Consequently, both intestinal and liver microsomes catalyze fentanyl metabolism and *N*-dealkylation by CYP3A4 is the principal active enzyme in both organs (Labroo et al., 1997). Hydroxylation occurs on the 2 or 3 position of the piperidine ring (a) (Figure 1), at the phenyl ring of the anilino moiety (b) (Figure 1), at the ethyl linker or the phenyl ring of the phenethyl moiety (c) (Figure 1), or along the amide alkyl chain (d) (Figure 1). The 4′-hydroxyfentanyl and other hydroxylated metabolites might be bioactive (Schneider and Brune, 1986), although the majority is believed to be inactive. The metabolite 4′-hydroxyfentanyl can undergo biotransformation via a second hydroxylation to allow a catechol that is then *O*-monomethylated to generate another metabolite. This reaction is probably catalyzed by the enzyme catechol-*O*-methyltransferase and presumably occurs at the 3′ position. This is technically a phase II metabolic product and can be detected in both hydrolyzed and non-hydrolyzed urine specimens due to its stability.

Minor metabolites such as hydroxypropionyl-fentanyl and hydroxypropionyl-norfentanyl are also created through different pathways without any relevant pharmacological activity. These metabolites have been detected in urine, stool and plasma (Bista et al., 2014).

Referring to despropionyl-fentanyl, another minor human metabolite, also known as 4-ANPP (Mahlke et al., 2014), results from carboxamide hydrolysis (Armenian et al., 2018).

Fentanyl is considered to be safer than morphine, in patients with liver and renal damage, because of a lack of metabolite accumulation (DePriest et al., 2015). Fentanyl activity can increase or decrease depending on genetic variations in the GI tract and in the liver, or through drugs which inhibit or induce CYP3A4. Fentanyl metabolism may be inhibited by macrolides, antifungal agents, and cimetidine (Bernard and Bruera, 2000). Serum fentanyl concentrations can vary significantly depending on liver function and the use of CYP3A4 inducers, therefore a model formula including these parameters has been provided, as a means to determine a transdermal fentanyl dose for the alleviation of cancer pain (Kokubun et al., 2012; Mercadante, 2015).

Bista and colleagues conducted a study to detect fentanyl and norfentanyl in plasma and saliva, showing that plasma and saliva had mean fentanyl concentrations of 0.785 and 3.335 μg/L, respectively. Similarly, in plasma and saliva the mean norfentanyl concentration was 0.53 and 0.517 μg/L, respectively. These data show that the concentration of fentanyl in saliva exceeds the concentration in plasma, suggesting an active transport into saliva. These data may in part be explained by the variable sample collection times with reference to time of dose, as distribution mechanisms will likely alter the saliva/plasma concentration ratio (Bista et al., 2014).

### Alfentanil, Sufentanil, Remifentanil

In humans, the other fentanyl analogs frequently used in anesthesia – alfentanil, sufentanil, and remifentanil – are extensively metabolized and just a little percentage of the dose is excreted in urine without metabolic transformation. Metabolites such as norsufentanil and noralfentanil seem to be pharmacologically inactive (Valaer et al., 1997; Skulska et al., 2004).

Alfentanil (*N*-{1-[2-(4-ethyl-5-oxo-4,5-dihydro-1H-tetrazol-1-yl)ethyl]-4-(methoxymethyl)-piperidin-4-yl}-*N*-phenylpropanamide) and sufentanil (*N*-{4-(methoxymethyl)-1-[2-(thiophen-2-yl)ethyl]piperidin-4-yl}-*N*-phenylpropanamide) are also principally metabolized in the liver via the CYP3A4 hepatic pathway, which generates the same *N*-dealkylated inactive metabolite, making a forensic distinction impossible when only this metabolite is detected (Armenian et al., 2018).

Compared to fentanyl, alfentanil has a smaller volume of distribution, greater binding to plasma proteins, less binding to red blood cells, a shorter elimination half-life, a slower total body clearance, and is less lipid soluble – characteristics which suggest that alfentanil would be an appropriate drug to give by continuous i.v. infusion (Fragen et al., 1983).

Sufentanil is metabolized by the liver and enterocytes of the small intestines, catalyzed by the cytochrome P450 enzyme system (Donk et al., 2018). Sufentanil metabolites are excreted in the urine. *N*-Dealkylation of sufentanil leads to mostly inactive metabolites such as the metabolites formed by oxidative *N*-dealkylation at the piperidine ring (norsufentanil) or the phenylpropanamide nitrogen (leading to *N*-phenylpropanamide) and by aromatic hydroxylation (Lavrijsen et al., 1990; Tateishi et al., 1996; Koyyalagunta, 2007). Norsufentanil retains some activity, whereas the oxidative *O*-demethylation product (demethylsufentanil) is active retaining about 10% of the activity of sufentanil. However, it is produced in small quantities only and therefore not clinically relevant. The extensive metabolism of sufentanil in the GI tract is responsible for the low bioavailability following oral administration, so if a patient accidentally swallows a sublingual tablet this will result in under-dosing. Although the absence of clinically relevant metabolites makes sufentanil an option in mild-to-moderate renal impairment, there is insufficient data in patients with severe renal impairment, and hence careful patient monitoring is advised (Donk et al., 2018).

Remifentanil {methyl 1-(3-methoxy-3-oxopropyl)-4-[phenyl(propanoyl) amino]piperidine-4-carboxylate} is metabolized directly in the plasma by non-specific esterases, a hugely active group of enzymes found in blood and tissues throughout the body, resulting in an ultra-short duration of action (Rosow, 1999; Panzer et al., 2009). It is the only fentanyl analog that is 95% metabolized in the blood and tissues by non-CYP enzymes, because of an easily accessible ester group allowing for rapid hydrolysis by circulating blood esterases (Armenian et al., 2018). Its primary metabolite is remifentanil acid (a carboxylic acid derivative, GR90291), which has negligible pharmacological activity. Therefore, although remifentanil acid is excreted by the kidneys, remifentanil’s action is not prolonged to a significant extent by renal injury or prolonged infusion in patients in intensive care (Panzer et al., 2009; Cascone et al., 2018). Experimental *in vivo* evaluations of the metabolic kinetics are presently not available (Cascone et al., 2018).

### Acetylfentanyl

Acetylfentanyl (*N*-Phenyl-*N*-[1-(2-phenylethyl)-4-piperidinyl] acetamide) is the acetyl amide analog of fentanyl. Relative potencies of several fentanyl analogs compared to fentanyl were evaluated by Higashikawa and Suzuki (2008a) in an animal study using the Litchfield–Wilcoxon test after peroral administration of diluted solutions of the fentanyl analogs to mice. ED_50_ and LD_50_ values obtained for acetylfentanyl were 0.021 and 9.3 mg/kg, respectively, suggesting about 30% of the analgesic potency of fentanyl.

In general, acetylfentanyl is metabolized in a similar way to fentanyl. Acetylfentanyl has a major primarily inactive metabolite, acetyl norfentanyl, produced by *N*-dealkylation via CYP450 enzymes (Patton et al., 2014; Watanabe et al., 2017). Melent’ev et al. (2015) investigated metabolism of acetylfentanyl in urine samples collected from fatal intoxication cases with this fentanyl analog. In this study, besides the proposed main metabolite acetyl norfentanyl and the deacetylated acetylfentanyl metabolite (4-ANPP), primarily hydroxylated acetylfentanyl metabolites and their phase II conjugates were detected. Hydroxylated metabolites of acetylfentanyl were also identified after incubation with hepatocytes (Kanamori et al., 2018b), including a 4′-hydroxy-3′-methoxy-metabolite which has also been found as a metabolite of fentanyl and was also detected by Melent’ev et al. (2015) in the death cases involving acetylfentanyl. In an additional work Kanamori et al. (2018a) determined the involvement of different CYP isoenzymes in the formation of the metabolites of acetylfentanyl described above. Moreover, Watanabe et al. (2017) identified 31 metabolites of acetylfentanyl in human hepatocytes and authentic human urine samples, including the β-hydroxy and 4′-hydroxy-3′-methoxy metabolite, and several other phase I and phase II metabolites formed via various pathways such as glucuronidation, sulfation, dihydroxylation, monohydroxylation, carbonylation, and dihydrodiol formation.

### Acryloylfentanyl

The fentanyl analog acryloylfentanyl (acrylfentanyl, *N*-Phenyl-*N*-[1-(2-phenylethyl)-4-piperidinyl]-acrylamide) differs from fentanyl only in dehydration in the 2,3-position of the propionylamide moiety. The competitive binding affinity of acryloylfentanyl was determined by Maryanoff et al. (1982) in rat brain using tritium-labeled naloxone. The IC_50_ value obtained was 1.4 nM and therefore similar to fentanyl (IC_50_ 1.6 nM). The analgesic properties of acryloylfentanyl were investigated by Essawi (1999) and it was found to be less potent than fentanyl (approximately 75% of fentanyl potency), but the analgesic effects persisted considerably longer. Though, the acrylamide moiety may lead to irreversible receptor binding and higher toxicity. However, LD_50_ values for acryloylfentanyl and fentanyl were 0.082 and 0.062 mg/kg, respectively, suggesting similar acute toxicity.

Similarly to fentanyl, acryloylfentanyl is lipophilic and expected to easily cross the blood-brain barrier. Distribution into fat and other tissues seems likely due to the presumably high volume of distribution (Ujváry et al., 2017). The metabolic pathway of acryloylfentanyl shows similarity with the pathways of fentanyl and acetylfentanyl. The main metabolites generated by human hepatocytes *in vitro* and of those detected in the urine in a few fatalities, caused by acryloylfentanyl, and their chemical structures were recently described by Watanabe et al. (2017). Overall, 14 biotransformation products, including major metabolites of acryloylfentanyl detected in human urine after hydrolysis of glucuronidated and/or sulfated phase II conjugates were identified in this work. The biotransformations involve an oxidative *N*-dealkylation, presumably catalyzed by cytochrome P450 (CYP450) enzymes, leading to the desphenethyl metabolite acryloylnorfentanyl which is biologically inactive. Furthermore, monohydroxylations were observed either at the alkyl linker of the phenylethyl moiety or at the piperidine ring. Dihydroxylation of the phenyl ring of the phenylethyl moiety resulting in a catechol structure followed by *O*-monomethylation were additional oxidative metabolic processes leading to similar metabolites as described for acetylfentanyl in the same work. Similar to fentanyl metabolism, amide hydrolysis (deacylation) results in a minor metabolite 4-ANPP, which is a common metabolite of fentanyl, acryloylfentanyl and several other fentanyl analogs. Acryloylfentanyl was also present in the urine of the deceased individuals.

With respect to acryloylfentanyl, the major human urinary metabolites identified *in vivo* (fatal cases) were acryloylnorfentanyl, as well as mono- and dihydroxylated derivatives and their conjugates (Watanabe et al., 2017).

### α-Methylfentanyl and (*cis*/*trans*)-3-Methylfentanyl

As one of the mono-methylated fentanyl derivatives, α-methylfentanyl (*N*-Phenyl-*N*-[1-(1-phenyl-2-propanyl)-4-piperidinyl]propanamide) carries the additional methyl group at the 1-position of the ethyl bridge of the phenethyl moiety. The diastereomeric pairs of enantiomers *cis*-3-methylfentanyl and *trans*-3-methylfentanyl carry the additional methyl group at the 3-position of the piperidine ring. The analgesic activity of these derivatives proved to be similar to fentanyl or higher. In a study of Higashikawa and Suzuki (2008a) α-methylfentanyl showed a very similar ED_50_ value as fentanyl (0.0058 and 0.0061 mg/kg, respectively). However, toxic effects occurred at significantly lower doses of α-methylfentanyl when compared to fentanyl (LD_50_ values 8.6 and 62 mg/kg, respectively). Van Bever et al. (1974) synthesized the different isomers of α-methylfentanyl and 3-methylfentanyl and investigated their relative analgesic potencies. ED_50_ values for α-methylfentanyl (0.0085 mg/kg) obtained in this work were in agreement with the reported values of Higashikawa and Suzuki (2008a), although fentanyl showed a higher value here (0.011 mg/kg). The (±)-*trans*-3-methylfentanyl enantiomers (ED_50_ 0.0094 mg/kg) showed about the same effective dose as α-methylfentanyl, but the (±)-*cis*-enantiomers turned out to be even more potent and exhibited a significant difference between the (+)- and (-)-enantiomer. The most potent isomer was (+)-*cis*-3-methylfentanyl (ED_50_ 0.00058 mg/kg) being about 20 times more potent than fentanyl, whereas the (-)-*cis*-isomer showed only 20% of the potency of fentanyl.

The first reports about detection of a metabolite of α-methylfentanyl were published by Gillespie (Gillespie et al., 1982), who found the hydrolysis product despropionyl-α-methylfentanyl in several biological samples of fatal intoxication cases. Higashikawa and Suzuki (2008b) investigated the metabolism of α-methylfentanyl in urine after administration to rats. The main metabolite formed by *N*-dealkylation in this study was norfentanyl, a metabolite shared with fentanyl. Furthermore, two metabolites in common with fentanyl were formed by further hydroxylation of the propionylamide moiety of norfentanyl [ω-hydroxypropionyl-norfentanyl and (ω-1)-hydroxypropionyl-norfentanyl]. However, four additional metabolites were identified enabling differentiation of α-methylfentanyl and fentanyl consumption resulting from mono- and dihydroxylation of α-methylfentanyl [ω-hydroxypropionyl-α-methylfentanyl, (ω-1)-hydroxypropionyl-α-methylfentanyl, *para*-hydroxy-phenyl-α-methylfentanyl and *para*-hydroxyphenyl-ω-hydroxypropionyl-α-methylfentanyl].

Investigations in rat performed by Sato et al. (2010) confirmed the findings of Higasikawa and Suzuki regarding the metabolic spectrum and demonstrated the time-course of metabolite excretion as well as the proportions of metabolites excreted in rat urine over a 96 h time period. Non-specific metabolites of α-methylfentanyl were detectable up to 72 h after administration, whereas the specific metabolites were completely eliminated after 48 h and accounted for only 2–3% of the total amount of metabolites excreted in urine.

First detection of single metabolites of the methylated fentanyl analog 3-methylfentanyl was reported by Hammargren and Henderson (1988) who detected the dealkylated metabolite nor-3-methylfentanyl in urine of suspected drug users. A systematic investigation of the metabolism of 3-methylfentanyl was done by Meyer et al. (2012) proposing a metabolic pathway for this fentanyl analog and reporting 9 phase I and 5 corresponding phase II metabolites in rat urine after drug administration. In accordance to Hammargren and Henderson, the main metabolite detected was nor-3-methylfentanyl formed by oxidative *N*-dealkylation. Further oxidation of this metabolite led to formation of hydroxypropionyl-nor-3-methylfentanyl and hydroxyphenyl-nor-3-methylfentanyl. In addition, mono- and dihydroxylations were observed primarily at the phenylethyl and the propionylamide moiety followed by either further oxidative reactions leading to a carboxy-propionyl metabolite or methylation of the 3,4-dihydroxyphenyl metabolite leading to a 3-methoxy-4-hydroxy metabolite in analogy to previously reported fentanyl and fentanyl analog metabolites. Furthermore, Meyer et al. (2012) also reported phase II glucuronic acid conjugates detected for hydroxylated metabolites.

### Isofentanyl

Isofentanyl (*N*-(1-benzyl-3-methylpiperidin-4-yl)-*N*-phenylpro-panamide) was clandestinely synthesized to circumvent 3-methylfentanyl regulation.

Meyer and collaborators identified isofentanyl together with 3-methylfentanyl phase I and phase II metabolites in rat urine (Meyer et al., 2012). Isofentanyl is an isomer of fentanyl and shares some main fragment ions in MS analysis. Metabolites such as the nor-metabolite can help to unequivocally prove uptake of this compound. For isofentanyl 11 phase I and 4 phase II metabolites were identified. The following metabolic steps could be postulated: *N*-dealkylation resulting in a common metabolite with 3-methylfentanyl (nor-3-methylfentanyl = nor-isofentanyl) followed by hydroxylations of the alkyl and/or aryl moiety, hydroxylation of the propionylamide side chain followed by oxidation to the corresponding carboxylic acid, and hydroxylations of the benzyl moiety followed by methylation resulting in the corresponding 3-methoxy-4-hydroxy metabolite. In addition, *N*-oxidation of isofentanyl was also observed. Some hydroxylated metabolites were partly excreted as glucuronides. Using recombinant human isoenzymes, CYP2C19, CYP2D6, CYP3A4, and CYP3A5 were found to be involved in the initial metabolic steps. The parent drugs could not be detected in urine. Their common nor-metabolite was suggested as a common target for urine screening for 3-methylfentanyl and isofentanyl. Targeting less abundant specific metabolites may enable differentiation of an uptake of either of the drugs (Meyer et al., 2012).

### Butyrfentanyl and Isobutyrfentanyl

Butyrfentanyl (*N*-Phenyl-*N*-[1-(2-phenylethyl)-4-piperidinyl] butanamide) belongs to the mono-methylated fentanyl derivatives carrying the additional methyl group at the ω-carbon of the propionyl amide resulting in a butyryl amide analog of fentanyl. Isobutyrfentanyl is the isomer carrying the additional methyl group at the α-carbon of the propionylamide moiety. Both compounds were included in the activity studies of Higashikawa and Suzuki (2008a). In a study from Alburges et al. (1992) the binding affinity of butyrfentanyl to the μ-opioid receptor was reported (*K*_i_ = 32 ± 4.1 nM), which is about 32-fold lower than the binding affinity of fentanyl (*K*_i_ = 1.03 ± 0.15 nM).

Metabolites of butyrfentanyl were detected and identified in a fatal poisoning described by Staeheli et al. (2016) with focus on the post mortem tissue distribution and redistribution, a phenomenon often observed when analyzing post mortem samples. The identified metabolites were norbutyrfentanyl, carboxybutyrfentanyl, hydroxybutyrfentanyl, and desbutyrfentanyl. In pursuit of elucidation of the metabolism of butyrfentanyl, blood and urine samples of the same fatal intoxication case in conjunction with *in vitro* studies producing phase I and phase II metabolites of butyrfentanyl were investigated by Steuer et al. (2017). Human liver microsomes and recombinant cytochrome P450 enzymes (CYP) were used for *in vitro* assays. Butyrfentanyl was shown to undergo extensive metabolism. In total, 36 metabolites were identified in this study. The postulated primary metabolic pathways were hydroxylations at the butanamide side chain (in two positions), the phenylethyl moiety and the piperidine ring, oxidative *N*-dealkylation, formation of *N*-oxides and hydrolysis of the acyl moiety. Besides that, combinations of these biotransformations and additional reactions were observed leading to, e.g., carboxylated metabolites by further oxidation of the ω-hydroxy-butanamide moiety or methylation of the 3,4-catechol moiety of dihydroxylated metabolites forming the respective 3-methoxy-4-hydroxy metabolites. Furthermore phase II conjugates were detected in the human post mortem samples for nine metabolites (eight glucuronic acid conjugates and one sulfate). The main metabolites detected in the *in vitro* studies, nor-butyrfentanyl, butyrohydroxy-butyrfentanyl and phenylethyl-hydroxy-butyrfentanyl, were not in agreement with the main metabolites detected in authentic biological samples. The main metabolites detected *in vivo* were carboxy-butyrfentanyl in blood and carboxy-butyrfentanyl, butyrohydroxy-butyrfentanyl and carboxy-phenylethyl-hydroxy-butyrfentanyl in urine. Initial screening experiments with the most relevant CYPs indicated that mainly CYP2D6 and 3A4 were involved in the primary metabolic steps. Therefore, variability of phenotypes regarding these enzymes may have an influence on the metabolic profile *in vivo*. As a strategy to reach maximum detectability it seems advisable to include metabolites formed by different pathways as targets into analytical methods.

### Carfentanil

Carfentanil [methyl 1-(2-phenylethyl)-4-[phenyl(propanoyl) amino]piperidine-4-carboxylate] is a member of the *N*-4 substituted fentanyl analogs carrying an additional methyl-carboxylate moiety at the 4-position of the piperidine ring. This group of fentanyl analogs turned out to be significantly more potent than their non-substituted analogs. Carfentanil is about 10,000 times more potent than morphine and shows 30–100 times the potency of fentanyl (Van Bever et al., 1976), thereby representing the most potent approved opioid drug. Receptor binding affinity and analgesic activity of this compound has been investigated extensively by many research groups, reporting ED_50_ values from 0.00032 nM up to 0.0017 (<0.01) nM and *K*_i_ values for the μ-opioid receptor of 0.024 nM up to 0.15 nM (Thompson et al., 1987; Costa et al., 1992; Maguire et al., 1992; Villemagne et al., 1994; Bi-Yi et al., 1999; Jewett and Kilbourn, 2004; Henriksen et al., 2005). Carfentanil is used in veterinary medicine as general anesthetic, for pain management, and to immobilize large animals (Kukanich and Papich, 2009).

Due to its extremely high potency studies assessing the metabolism of carfentanil in humans have not been performed yet and it seems unlikely that they would be approved by an Ethics Committee. Though, metabolites of carfentanil have only been detected in fatal intoxication cases so far, the most well-known case being the Moscow Theater hostage-taking (Riches et al., 2012). Riches et al. (2012) detected the *N*-dealkylated metabolite norcarfentanil in a donated urine sample. Norcarfentanil is a common (minor) metabolite of the fentanyl analog remifentanil. First and only studies assessing the metabolic pathways of carfentanil were performed by Feasel et al. (2016) using metabolism predictions software (Molecular Discovery’s MetaSite software and Simulations Plus’s ADMET Predictor) for first *in silico* prediction and human liver microsomes and hepatocytes as *in vitro* models.

In total, 12 metabolites were identified for carfentanil, 11 phase I metabolites and 1 phase II conjugate as glucuronide. The following metabolic reactions or combinations of these were observed: oxidative *N*-dealkylation, ester hydrolysis, hydroxylation and *N*-oxide formation. The most abundant metabolites reported were formed by *N*-dealkylation partly followed by ester hydrolysis or hydroxylation. Hydroxylations occurred at the propionylamide side chain, at the phenylethyl moiety or at the piperidine ring resulting in formation of eight hydroxylated metabolites, and five of them showed an additional biotransformation (ester hydrolysis, *N*-oxide formation or glucuronidation) or were further oxidized to ketones. Three *N*-oxide metabolites were reported with minor abundances, formed by oxidation of either the piperidine nitrogen or the anilino-nitrogen linked in the amide moiety. In contrast to studies concerning the metabolism of other fentanyl analogs so far, no amide hydrolysis metabolites or hydroxy-methoxy metabolites have been reported in this study.

### Alicyclic Fentanyl Analogs: Cyclopropylfentanyl, Cyclobutylfentanyl Cyclopentylfentanyl, Cyclohexylfentanyl and 2,2,3,3-Tetramethylcyclopropyl-Fentanyl

This subgroup of newly emerging fentanyl analogs structurally differs in the aliphatic amide linked moiety, which is substituted by an aliphatic cyclic moiety in these compounds. Concerning the receptor binding affinities and potencies only cyclopropylfentanyl has been evaluated so far. *In vitro* studies using chinese hamster ovary (CHO) and rat cell preparations expressing the three types of opioid receptors were used for determination of binding affinities. Cyclopropylfentanyl binds selectively to the μ-opioid receptor (vs. [^3^H]-DAMGO) with *K*_i_ values of 0.088 ± 0.027 nM for the μ-opioid receptor as well as 59.4 ± 3.0 nM and 36 ± 10 nM for the δ- and κ-opioid receptors, respectively. EC_50_ values were determined employing a [^35^S]GTPγS binding assay, resulting in 10.8 ± 2.7 nM for cyclopropylfentanyl at the μ-opioid receptor compared to 32 ± 11 nM for fentanyl, showing a more or less similar (about threefold higher) potency to fentanyl (Drug Enforcement Administration–Veterans Affairs (DEA-VA) Interagency Agreement, 2017; European Monitoring Centre for Drugs Drug Addiction [EMCDDA], 2018b). For the other alicyclic analogs no literature on receptor binding affinities and potencies is available yet. Theoretically, these analogs may imitate the steric requirements for receptor binding of fentanyl. They probably show similar or lower potency than fentanyl, in analogy to butyr- and valerylfentanyl which have been reported to be less potent.

A study investigating metabolism of this group of compounds has been published very recently by Åstrand et al. (2018) using human hepatocytes. Seven metabolites were identified for cyclopropylfentanyl, and the most abundant metabolite was norcyclopropylfentanyl formed by oxidative *N*-dealkylation. Other metabolic reactions observed were monohydroxylation, dihydroxylation followed by subsequent methylation, dihydrodiol and *N*-oxide formation. The glucuronic acid conjugate of the most intense hydroxy metabolite (hydroxylated at the piperidine ring) was detected as well. Hydroxylation of the cyclopropyl moiety or amide hydrolysis has not been detected in this study. The main metabolite norcyclopropylfentanyl has also been detected by Maher et al. (2018) in urine samples of two death cases and Palaty et al. (2018) in several urine samples of patients with a substance use disorder from two canadian provinces.

The major metabolites detected for cyclobutylfentanyl by Åstrand et al. (2018) were also *N*-dealkylation and, in contrast to cyclopropylfentanyl, hydroxylation of the cyclobutyl moiety and amide hydrolysis leading to a metabolite found in common with fentanyl, 4-ANPP. Further mono- and dihydroxylated metabolites were identified, mainly hydroxylated at the cyclobutyl moiety, the piperidine ring or the phenylethyl moiety.

In agreement with findings of cyclobutylfentanyl, the most abundant metabolites found were hydroxylations of the cyclopentyl moiety and nor-cyclopentylfentanyl. Moreover, another monohydroxlated metabolite (at the piperidine ring) and two monohydroxylated normetabolites (both at the cyclopentyl ring), a dihydroxylated metabolite (at the piperidine ring and the cyclopentyl ring), the amide hydrolysis product 4-ANPP and two further oxidation products (*N*-oxide and carbonyl metabolite) were formed to a minor extent in this assay.

Incubation of cyclohexylfentanyl with hepatocytes mainly led to the amide hydrolysis product 4-ANPP, norcyclohexylfentanyl and two monohydroxlated metabolites (both modified at the cyclohexyl moiety). Again, further hydroxylated metabolites were detected, comprising monohydroxlation, dihydroxylation, and hydroxylations in a second metabolic step primarily at the cyclohexyl and the piperidine ring.

Substitution of the amide linked alkyl chain with a 2,2,3,3-tetramethylcyclopropyl moiety seemed to steer metabolic reactions to this part of the molecule. Except for the nor-2,2,3,3-tetramathylcyclopropylfentanyl metabolite, which was formed to a minor extent, all metabolites showed at least one biotransformation of the 2,2,3,3-tetramethylcyclopropyl moiety. Monohydroxylations and dihydroxylations and subsequent further oxidation steps resulting in the formation of, e.g., carboxylic acids have been reported by Åstrand et al. (2018).

### 4-Fluoroisobutyrfentanyl (4F-iBF, *Para*-Fluoroisobutyrfentanyl)

4-fluoroisobutyrfentanyl (4-fluoro-isobutylfentanyl (*N*-(4-fluorophenyl)-2-methyl-*N*-[1-(2-phenylethyl)-4-piperidinyl]-propanamide) is one of the fluorinated fentanyl analogs that emerged on the NPS drug market recently. For this analog no data on binding affinity and selectivity to the μ-opioid receptor is available. The potency of 4F-iBF might be similar or lower than the potency of butyrfentanyl/isobutyrfentanyl, following the evaluation of fluorinated derivatives by Higashikawa and Suzuki (2008a). Metabolism of 4F-iBF was investigated by Watanabe et al. (2017) using hepatocyte incubates and analyzing authentic urine samples. In total, 17 metabolites were found and the following biotransformations were observed: *N*-dealkylation, monohydroxylations, dihydroxylations and subsequent methylation and glucuronidation, dihydrodiol formation, amide hydrolysis, carbonylation and carboxylation. Nine metabolites were identified in the hepatocyte assay. The most abundant ones were nor-4F-iBF, and two hydroxylated metabolites (at the piperidine ring or the phenylethyl moiety). Analysis of the urine samples after conjugate cleavage revealed 11 metabolites, resulting in a similar metabolic profile as obtained from the hepatocyte incubation assay, although the hydroxy-methoxy metabolite was more dominant in the authentic urine sample. Two additional glucuronic acid conjugates were detected when analyzing the urine without hydrolysis prior to extraction.

### Furanylfentanyl

Binding affinity studies on furanylfentanyl have been performed *in vitro* using CHO and rat cell preparations expressing the three types of opioid receptors. Furanylfentanyl binds selectively to the μ-opioid receptor (vs. [^3^H]-DAMGO) with *K*_i_ values of 0.028 ± 0.008 nM for the μ-opioid receptor as well as 54 ± 15 nM and 59.2 ± 6.4 nM for the δ- and κ-opioid receptors, respectively. *In vitro* EC_50_ values were determined employing a [^35^S]GTPγS binding assay, resulting in 2.52 ± 0.46 nM for furanylfentanyl compared to 17.9 ± 4.3 nM for fentanyl at the μ-opioid receptor, suggesting a sevenfold higher potency for furanylfentanyl over fentanyl (Drug Enforcement Administration–Veterans Affairs (DEA-VA) Interagency Agreement, 2016; European Monitoring Centre for Drugs Drug Addiction [EMCDDA], 2017). In the patent literature of furanylfentanyl an *in vivo* ED_50_ value (0.02 mg/kg) was reported after i.v. administration to mice, but comparative data of fentanyl or morphine was not reported (Huang et al., 1986).

Goggin et al. (2017) identified four metabolites of furanylfentanyl after analyzing 500 urine samples of opioid intoxication cases. The most pronounced metabolites detected in 42 out of 51 cases positive for furanylfentanyl was the hydrolysis product 4-ANPP and its sulfate conjugate. Moreover, a very unique metabolite formed by dihydrodiol formation of the heterocyclic furanyl moiety was detected in 86% of the cases. The *N*-dealkylated metabolite norfuranylfentanyl was detected in only four of the furanylfentanyl positive cases indicating that substitution of the amide linked moiety of the fentanyl analogs to a furanyl-carboxamide shifts the metabolic profile of this compound toward a hydrolytic reaction and biotransformation of the furanyl moiety. In accordance with these findings, Mohr et al. (2016) detected 4-ANPP in five out of eight fatal intoxications with furanylfentanyl and Martucci et al. (2018) reported detection and distribution of the hydrolysis metabolite 4-ANPP in various tissues of a fatal furanylfentanyl intoxication case. In total, 17 and 14 phases I and II metabolites of furanylfentanyl were identified in a more detailed *in vitro* approach by Richeval et al. (2017) and Watanabe et al. (2017) using human liver microsomes and hepatocytes. In contrast to the findings of Goggins and Martucci, the spectrum of metabolic reactions in these *in vitro* studies comprised several hydroxylations, *N*-oxide formation and glucuronidation besides the already mentioned amide hydrolysis (plus sulfate conjugation), dihydrodiol formation and *N*-dealkylation. The most abundant *in vitro* metabolites reported by both authors were the hydrolysis product 4-ANPP, a dihydrodiol metabolite and norfuranylfentanyl. Additionally, a metabolite formed by oxidative ring-opening of the furanyl ring (and further oxidation to a carboxylic acid) was reported by both groups. Since metabolism of furanylfentanyl has been studied by a couple of research groups it can be said, that *N*-dealkylation which often leads to main metabolites of fentanyl and fentanyl analogs *in vitro* and *in vivo*, plays a minor role in the metabolism of furanylfentanyl, whereas amide hydrolysis and oxidative transformations of the furanyl moiety (such as dihydrodiol formation) are major biotransformation steps seen both *in vitro* and *in vivo* for this compound.

### Methoxyacetylfentanyl

Methoxyacetylfentanyl is one of the numerous newly emerged fentanyl analogs differing from fentanyl by the modification of the *N*-acyl moiety. Structure activity relationships of methoxyacetylfentanyl and several other alkyloxy derivatives were investigated by Bagley et al. (1991) reporting an ED_50_ value of 0.053 mg/kg for methoxyacetylfentanyl. Compared to the ED_50_ of 0.018 mg/kg for fentanyl, about 30% of the potency of fentanyl can be assumed for this compound.

Metabolism of methoxyacetylfentanyl (2-methoxy-*N*-(1-phenethylpiperidin-4-yl)-*N*-phenylacetamide) was first examined *in vitro* using a human liver microsomal preparation (European Monitoring Centre for Drugs Drug Addiction [EMCDDA], 2018a). A main metabolic step for methoxyacetylfentanyl appears to be *O*-demethylation leading to 2-hydroxyacetamide metabolite. Further metabolic reactions were *N*-dealkylation, hydroxylations of the piperidine ring and the phenylethyl side chain, *N*-oxidation, as well as amide hydrolysis to 4-ANPP.

Mardal et al. (2018) investigated the *in vitro* and *in vivo* metabolic profiles of methoxyacetylfentanyl in the context of three case reports on deaths related to methoxyacetylfentanyl and by applying an additional *in vitro* study using human hepatocytes. A total of 10 methoxyacetylfentanyl metabolites were identified in hepatocyte incubates and biological samples. The metabolic pathways comprised mono- and dihydroxylations (at the phenylethyl ring or the anilinophenyl ring), *N*-dealkylation, *O*-demethylation, amide hydrolysis and combinations thereof as well as *O*-glucuronidation of the *O*-demethylated metabolite. The main metabolites both detected *in vitro* and *in vivo* were the *O*-demethylated metabolite and the hydrolysis product 4-ANPP. The findings of this study were consistent with unpublished data provided to the EMCDDA for risk assessment of methoxyacetylfentanyl (European Monitoring Centre for Drugs Drug Addiction [EMCDDA], 2018a).

### Ocfentanil

This fentanyl analog has been developed and patented by Huang et al. (1986) and was evaluated for clinical application. Ocfentanil (*N*-(2-fluorophenyl)-2-methoxy-*N*-[1-(2-phenylethyl)-4-piperidinyl]acetamide) is the *ortho*-fluorinated analog of methoxyacetylfentanyl and has also been subject to the studies of Bagley et al. (1991). They determined an ED_50_ value of 0.0077 mg/kg for ocfentanil using the mouse hot plate test for evaluation of analgesic effects. Compared to fentanyl (0.018 mg/kg) the potency can be estimated to be about 2.5 times higher than for fentanyl. At the same time, ocfentanil showed less respiratory depression in animal studies. Fletcher et al. (1991) investigated dose-dependent pharmacologic effects in humans but were not able to draw conclusions from the study regarding a benefit of ocfentanil over fentanyl.

Allibe et al. (2018) performed metabolism studies on ocfentanil using human liver microsomes in addition to metabolism profiling in post mortem samples of a fatal intoxication case. Ocfentanil was found in all biological samples except nasal swab and concentrations were similar in peripheral blood and cardiac blood (Allibe et al., 2018). This observation is in contrast to results in two previously reported fatalities which observed significant deviations of the cardiac/peripheral blood concentration ratio (Coopman et al., 2016; Dussy et al., 2016).

Four metabolites were detected *in vitro* by Allibe et al. (2018) formed by hydroxylation (at the phenylethyl moiety), *O*-demethylation and combination of both reactions as well as the conjugation product of the *O*-demethyl metabolite with glucuronic acid. The main metabolite *in vitro* and *in vivo* clearly was *O*-demethyl ocfentanil, presumably even exceeding quantities of the parent compound (when comparing peak areas). In contrast, commonly seen biotransformations such as *N*-dealkylation and amide hydrolysis have not been detected in this work, suggesting that these metabolic reactions only play a minor role in metabolism of ocfentanil.

### *Ortho*-Fluorofentanyl

*Ortho*-fluorofentanyl (*N*-(2-fluorophenyl)-*N*-[1-(2-phenylethyl)-4-piperidinyl]-propanamide) is a fluorinated fentanyl derivative. No data on receptor binding is available for this compound so far. However, the *para*-substituted analog was included in the studies performed by Higashikawa and Suzuki (2008a) and showed about 30% the potency of fentanyl determined by ED_50_ values. The LD_50_ values of 9.3 mg/kg for *p*-fluorofentanyl compared to 63 mg/kg for fentanyl indicate a higher toxicity of the fluorinated compound. The only study reporting metabolite identification of *ortho*-fluorofentanyl so far was a case report from Denmark by Andreasen et al. (2017). They detected the *N*-dealkylation product *ortho*-fluoro-norfentanyl in blood by HRMS techniques. Other potential metabolites like hydroxy-*ortho*-fluorofentanyl, hydroxy-*ortho*-fluoro-norfentanyl or the hydrolysis product *ortho*-fluoro-despropionylfentanyl were not detected in the authentic case sample. However, a urine sample was not part of the investigation, which could be the reason for the limited number of detected metabolites. The amide hydrolysis product *ortho*-fluoro-despropionylfentanyl has been reported to the EMCDDA as a fentanyl analog marketed independently, but further data on this compound is not available so far. A case report from Helland et al. (2017) focuses on the identification of *ortho*-fluorofentanyl and problems with the distinction of stereo-isomers. In this work the authors emphasize the necessity of integrating fluorinated analogs into general analytical screening procedures.

### Tetrahydrofuranylfentanyl

The binding affinity of tetrahydrofuranylfentanyl (THFF, *N*-phenyl-*N*-[1-(2-phenylethyl)piperidin-4-yl]oxolane-2-carbo-xamide) was determined by the United States Drug Enforcement Administration (DEA) using CHO and rat cell preparations for opioid receptor expression. *K*_i_ values for THFF were 0.95 ± 0.32 nM (μ-OR vs. [^3^H]-DAMGO), compared to 741 ± 44 nM (vs. [^3^H]-U-69593) and 1,730 ± 260 nM (vs. [^3^H]-DPDPE) for the δ- and κ-opioid receptors, respectively, showing high selectively for the μ-opioid receptor. EC_50_ values were determined *in vitro* employing an [^35^S]GTPγS binding assay and resulted in 89 ± 16 nM for THFF at the μ-opioid receptor. The authors report a lower potency compared to fentanyl for this compound (European Monitoring Centre for Drugs Drug Addiction [EMCDDA], 2018c).

Data provided to the EMCDDA for risk assessment of THFF propose *N*-dealkylation to be the predominant metabolic step for THFF in human liver microsomal preparations, as in the case of fentanyl. Hydroxylation of the piperidine ring and the phenylethyl side chain, *N*-oxidation and amide hydrolysis to 4-ANPP were also observed (European Monitoring Centre for Drugs Drug Addiction [EMCDDA], 2018c).

Metabolic profiling of THFF was performed by Krotulski et al. (2018) to assist analytical identification of THFF in a fatality. Overall, seven metabolites were identified *in vitro* for THFF using pooled human liver microsomes. The hydroxylated metabolite species produced multiple, indistinguishable signals for hydroxylations at the tetrahydrofuranyl ring or the phenylethyl moiety. One of the major metabolites *in vitro* was nortetrahydrofuranylfentanyl formed by *N*-dealkylation, which proofed to be an applicable biomarker for THFF ingestion in biological samples. The hydroxylated species were also prominently detected in post mortem blood and urine samples. The hydrolysis product 4-ANPP was not unequivocally identified as a metabolite in this study (for analytical reasons), but may be considered as a possible minor metabolite since another hydroxylated metabolite (hydroxyl-4-ANPP) was also identified. Additionally, a biotransformation product presumably formed by oxidation of the tetrahydrofuranyl moiety and subsequent ‘internal hydrolysis’ under ring-opening was identified (Krotulski et al., 2018).

A short summary of the reviewed fentanyl analogs and their main metabolites (and metabolic pathways) described in the literature and estimated relative potencies (compared to fentanyl) are listed in Table 1.

**Table 1:** Summary of the reviewed fentanyl analogs and their metabolites and metabolic pathways.

Compounds	Detected metabolites (metabolic pathways)	Estimated relative potencies to fentanyl
Alfentanil	Noralfentanil (*N*-dealkylation)	Approximately 0.3
Sufentanil	Norsufentanil and *N*-phenylpropanamide (*N*-dealkylation), demethylsufentanil (*O*-demethylation), hydroxy metabolites	Approximately 10
Remifentanil	Remifentanil acid (ester hydrolysis)	Approximately 1
Acetylfentanyl	Acetyl norfentanyl (*N*-dealkylation), 4-ANPP (amide hydrolysis), β-hydroxyacetylfentanyl and further hydroxy metabolites, 4’-hydroxy-3’-methoxy-acetylfentanyl (dihydroxylation + methylation) and phase II conjugates	0.3
Acryloylfentanyl	Acryloylnorfentanyl (*N*-dealkylation), 4-ANPP (amide hydrolysis), β-hydroxyacryloylfentanyl and further hydroxy metabolites, 4’-hydroxy-3’-methoxy-acryloylfentanyl (dihydroxylation + methylation) and phase II conjugates	Approximately 0.75
α-Methylfentanyl	Norfentanyl (*N*-dealkylation), Despropionyl-α-methylfentanyl (amide hydrolysis), alkyl/aryl hydroxy metabolites	Approximately 1
*Cis*-3-methylfentanyl *Trans*-3-methylfentanyl	Nor-3-methylfentanyl (*N*-dealkylation), alkyl/aryl hydroxy metabolites, carboxypropionyl-3-methylfentanyl (hydroxylation + oxidations), 4′-hydroxy-3′-methoxy-3-methylfentanyl (dihydroxylation + methylation) and phase II conjugates	20 (+) isomer 0.2 (-) isomer Approximately 1
Isofentanyl	Nor-3-methylfentanyl (*N*-dealkylation), alkyl/aryl hydroxy metabolites, carboxypropionyl-isofentanyl (hydroxylation + oxidations), 4′-hydroxy-3′-methoxy-isofentanyl (dihydroxylation + methylation), *N*-oxide formation and phase II conjugates	n.a.
Butyrfentanyl	Norbutyrfentanyl (*N*-dealkylation), carboxybutyrfentanyl (hydroxylation + oxidations), 4-ANPP (amide hydrolysis), alkyl/aryl hydroxy metabolites, 4′-hydroxy-3′-methoxy-butyrfentanyl (dihydroxylation + methylation), *N*-oxide formation and phase II conjugates	0.03–0.13
Isobutyrfentanyl	n.a.	0.13
Carfentanil	Norcarfentanil (*N*-dealkylation), alkyl/aryl hydroxy metabolites, carfentanil acid (ester hydrolysis), keto-carfentanil (hydroxylation + oxidation), *N*-oxide formation and phase II conjugates	30–100
Cyclopropylfentanyl	Norcyclopropylfentanyl (*N*-dealkylation), hydroxylations, dihydrodiol and *N*-oxide formation	3
Cyclobutylfentanyl	Norcyclobutylfentanyl (*N*-dealkylation), mainly alkyl hydroxy metabolites, 4-ANPP (amide hydrolysis), *N*-oxide and ketone formation	n.a.
Cyclopentylfentanyl	Norcyclopentylfentanyl (*N*-dealkylation), mainly alkyl hydroxy metabolites, 4-ANPP (amide hydrolysis), *N*-oxide and ketone formation	n.a.
Cyclohexylfentanyl	Norcyclohexylfentanyl (*N*-dealkylation), mainly alkyl hydroxy metabolites, 4-ANPP (amide hydrolysis), *N*-oxide and ketone formation	n.a.
2,2,3,3-Tetramethyl-cyclopropylfentanyl	Mainly alkyl hydroxy metabolites, Nor-2,2,3,3-tetramethylcyclopropylfentanyl (*N*-dealkylation), carboxy-2,2,3,3-tetramethylcyclopropylfentanyl (hydroxylation + oxidations)	n.a.
4-Fluoroisobutyrfentanyl	Nor-4-fluoroisobutyrfentanyl (*N*-dealkylation), alkyl/aryl hydroxy metabolites, 4-ANPP (amide hydrolysis), 4′-hydroxy-3′-methoxy-4-fluoroisobutyrfentanyl (dihydroxylation + methylation), dihydrodiol and ketone formation, carboxy-4-fluoroisobutyrfentanyl (hydroxylation + oxidations) and phase II conjugates	n.a.
Furanylfentanyl	Furano-dihydrodiol formation, 4-ANPP (amide hydrolysis), norfuranylfentanyl (*N*-dealkylation), alkyl/aryl hydroxy metabolites, ring opening of the furanyl ring and phase II conjugates	7
Methoxyacetylfentanyl	Demethylmethoxyacetylfentanyl (*O*-demethylation), 4-ANPP (amide hydrolysis), normethoxyacetylfentanyl (*N*-dealkylation), alkyl/aryl hydroxy metabolites and phase II conjugates	0.3
Ocfentanil	Demethylocfentanil (*O*-demethylation), alkyl/aryl hydroxy metabolites and phase II conjugates	2.5
*Ortho*-Fluorofentanyl	Nor-*ortho*-fluorofentanyl (*N*-dealkylation)	n.a.
Tetrahydrofuranylfentanyl	Nortetrahydrofuranylfentanyl (*N*-dealkylation), alkyl/aryl hydroxy metabolites, ring opening of the tetrahydrofuranyl ring and 4-ANPP (amide hydrolysis)	Approximately 0.2

*Estimated relative potencies compared to fentanyl (set to 1) are also given (n.a., no data available).*

A number of further fentanyl analogs have been reported to the EMCDDA (mainly referring to seizures by police or customs authorities or intoxication cases) and were included into the literature search. However, no data on potency, receptor binding or metabolism was available yet. For the sake of completeness these compounds will be listed here in alphabetical order:

2-fluoroisobutyrfentanyl, 2-methyl-acetylfentanyl, 3-methyl-crotonylfentanyl (senecionyl fentanyl), 3-fluorofentanyl, 3-phenylpropanoylfentanyl, 4-chloroisobutyrfentanyl, 4-fluoro-butyrfentanyl, 4-fluoro-cyclopropylbenzylfentanyl, 4-fluoro-fentanyl, 4-fluoroisobutyrfentanyl *N*-benzyl analog, 4-methox-ybutyrfentanyl, α-methylfentanyl butanamide analog (2-methyl-*N*-phenyl-*N*-[1-(1-phenyl-propan-2-yl)piperidine-4-yl]propa-namide), acetyl benzylfentanyl, benzodioxolefentanyl, benzoyl-benzylfentanyl, benzoylfentanyl, benzylfentanyl, crotonyl-fentanyl, furanylbenzylfentanyl, furanylethylfentanyl, furanyl-fentanyl-3carboxamide isomer, thiophenefentanyl and valerylfentanyl.

## Author Contributions

RS, SP, RP, AT, FB, VA, and MW searched for bibliographic material, drafted different chapter of the manuscript, and contributed substantially to manuscript intellectual content and revision.

## Conflict of Interest Statement

The authors declare that the research was conducted in the absence of any commercial or financial relationships that could be construed as a potential conflict of interest.
